# Uptake of HIV testing and outcomes within a Community-based Therapeutic Care (CTC) programme to treat Severe Acute Malnutrition in Malawi: a descriptive study

**DOI:** 10.1186/1471-2334-8-106

**Published:** 2008-07-31

**Authors:** Paluku Bahwere, Ellen Piwoz, Marthias C Joshua, Kate Sadler, Caroline H Grobler-Tanner, Saul Guerrero, Steve Collins

**Affiliations:** 1 Valid International, Unit 9, Standingford House, 26 Cave Street, Oxford, OX4 1BA, UK; 2 Academy for Educational Development, Washington DC, USA; 3 Ministry of Health, Dowa District Hospital, PO Box 25, Dowa, Malawi

## Abstract

**Background:**

In Malawi and other high HIV prevalence countries, studies suggest that more than 30% of all severely malnourished children admitted to inpatient nutrition rehabilitation units are HIV-infected. However, clinical algorithms designed to diagnose paediatric HIV are neither sensitive nor specific in severely malnourished children. The present study was conducted to assess : i) whether HIV testing can be integrated into Community-based Therapeutic Care (CTC); ii) to determine if CTC can improve the identification of HIV infected children; and iii) to assess the impact of CTC programmes on the rehabilitation of HIV-infected children with Severe Acute Malnutrition (SAM).

**Methods:**

This community-based cohort study was conducted in Dowa District, Central Malawi, a rural area 50 km from the capital, Lilongwe. Caregivers and children admitted in the Dowa CTC programme were prospectively (Prospective Cohort = PC) and retrospectively (Retrospective Cohort = RC) admitted into the study and offered HIV testing and counseling. Basic medical care and community nutrition rehabilitation was provided for children with SAM. The outcomes of interest were uptake of HIV testing, and recovery, relapse, and growth rates of HIV-positive and uninfected children in the CTC programme. Student's t-test and analysis of variance were used to compare means and Kruskall Wallis tests were used to compare medians. Dichotomous variables were compared using Chi^2 ^analyses and Fisher's exact test. Stepwise logistic regression with backward elimination was used to identify predictors of HIV infection (α = 0.05).

**Results:**

1273 and 735 children were enrolled in the RC and PC. For the RC, the average age (SD) at CTC admission was 30.0 (17.2) months. For the PC, the average age at admission was 26.5 (13.7) months. Overall uptake of HIV testing was 60.7% for parents and 94% for children. HIV prevalence in severely malnourished children was 3%, much lower than anticipated. 59% of HIV-positive and 83% of HIV-negative children achieved discharge Weight-For-Height (WFH) ≥ 80% of the NCHS reference median (p = 0.003). Clinical algorithms for diagnosing HIV in SAM children had poor sensitivity and specificity.

**Conclusion:**

CTC is a potentially valuable entry point for providing HIV testing and care in the community to HIV infected children with SAM.

## Background

Access to prophylactic cotrimoxazole and to timely initiation of antiretroviral therapy can tremendously improve the survival of HIV infected children [1-5]. HIV testing is the "gateway" or first step to introducing infected children to these interventions[6]. Unfortunately, the coverage of HIV testing still remains low in most resource constrained countries, particularly for children, and existing pediatric clinical algorithms have low sensitivity for detecting HIV, particularly among the severely malnourished [7,8]. Thus, most HIV-infected children remain undiagnosed, even in households with an adult already on antiretroviral treatment (ART)[9].

Reasons for low coverage of pediatric testing are varied but include high postnatal dropout from Prevention of Mother-to-Child Transmission (PMTCT) programmes, and relatively few testing facilities, especially in rural areas [10,11]. In 2005, Malawi had an estimated 91,000 HIV infected children (≤ 14 years) [12]. Among them, the cumulative number on ART by March 2006 was 2718, representing only 3.0% of all infected children and 5.8% of patients on ART in Malawi to date [12].

The HIV epidemic is contributing to increased child mortality and severe malnutrition throughout Africa [13,14]. In Malawi and elsewhere where HIV is highly prevalent, studies suggest that 30% or more of all children admitted to inpatient nutrition rehabilitation units are HIV-infected [13,15-17]. A prior study in Malawi demonstrated that many HIV-infected children with severe acute malnutrition (SAM) can achieve an adequate Weight-For-Height (WFH) with appropriate therapeutic feeding, although recovery times are significantly longer and mortality is much higher compared to HIV-uninfected children [18]. Unfortunately in Africa, the low coverage of both HIV testing and therapeutic feeding and the multiple factors preventing caregivers bringing children to hospital and remaining with them for extended periods [7,19,20] means that the majority of all HIV-infected African children do not receive any nutritional care. Thus, it is important to find innovative ways of identifying HIV-infected children, especially those from remote areas, to allow them to benefit from cotrimoxizole prophylaxis, nutritional care, and ARV treatment in developing countries.

Community-based Therapeutic Care (CTC) is an approach for managing SAM in children < 5 years that provides care close to where people live and focuses on early detection of SAM through community mobilization [21]. Children with appetite and without complications are managed directly in the community in an Outpatient Therapeutic Programme (OTP) and provided with routine medical care and nutrient-dense, pathogen resistant Ready-to-Use Therapeutic Foods (RUTF) for nutritional rehabilitation [22] on a weekly basis. Children with generalized severe oedema, anorexia and medical complications are treated at inpatient facilities according to national protocols, until they are stabilized and appetite has returned and they are able to rejoin the OTP [23,24]. Children are discharged from the OTP when they achieve WFH ≥ 80%. Previous evaluations show that CTC achieves high coverage and has lower default and mortality rates compared to traditional therapeutic feeding programs [25,26]. Throughout Africa, home-based care programs are providing support for HIV-affected households. We undertook this study to identify appropriate ways of diagnosing HIV among malnourished children and to assess the feasibility and outcome of treating severely malnourished HIV-infected children in a community based program. This paper presents the uptake of HIV testing when integrated within an ongoing CTC programme, the comparative outcomes of HIV-infected and uninfected malnourished children enrolled in the programme and the diagnostic value of anthropometric and other admission characteristics for identifying children at risk of HIV where diagnostic testing is not available.

## Methods

The study was carried out from December 2002 to May 2005 in the Dowa district of central Malawi, where antenatal and adult HIV prevalence were estimated to be 9.8% and 6.4%, respectively [27,28] Voluntary HIV Counselling and Testing (VCT) was offered to caregivers and children who were enrolled or had recently graduated from a CTC programme run by the Ministry of Health and the non-governmental organization Concern Worldwide. Clinical records were reviewed to assess our primary outcomes: HIV testing uptake and test results and indices of nutritional recovery in HIV-infected and uninfected children. Two groups of caregivers were invited to participate: 1) those who were discharged from CTC prior to VCT introduction (retrospective cohort), and 2) those who entered CTC after testing was introduced (prospective cohort).

### Retrospective Cohort (RC)

Traditional leaders, Health Surveillance Assistants (HSAs), and community volunteers were responsible for locating recent programme graduates using CTC discharge records. Caregivers were visited at home to explain the study objectives and procedures and to invite them to bring their children to the next recruitment day. A verbal autopsy was carried out for children who had died since leaving the programme [27]. Only families of children presently residing in the geographic catchment areas of the 17 health centres providing CTC in Dowa district were contacted. At recruitment, VCT was offered to children and their caregivers. Pre and post-test counselling was carried out by trained nurses and HSAs in accordance with guidelines from the Malawi Ministry of Health for HIV testing and counselling of adults and children < 13 years [28].

Outcomes of interest from the RC include: 1) HIV testing uptake and outcome; 2) nutritional recovery whilst in the CTC programme (weight gain, change in Mid Upper Arm Circumference, time to recovery); and 3) mortality and change in nutritional status from CTC discharge to recruitment. Clinical data were extracted from an electronic database and individual programme monitoring cards.

### Prospective cohort (PC)

Procedures were similar for the PC children except that VCT was offered at admission to the programme. All PC children were treated according to standard CTC protocols and children whose caregivers chose not to join the study were not treated any differently. Children were recruited into the study from 29/11/2004 to 09/04/2005 corresponding to the 2004/2005 hunger period (~70% of annual admissions are usually observed during the hunger season). The outcomes of interest of the PC were the same than that of the RC with 2 exceptions: 1) we could calculate mortality and programme default (children who left the programme prior to achieving > 85% of median WFH); 2) data on follow up after discharge were unavailable.

### Data collection

At admission to the study, a clinical assessment and history was obtained for each child by one of three trained nurses using a structured data collection tool. The history included ascertainment of the signs and symptoms used for clinical diagnosis of paediatric HIV in algorithms used by WHO [29,30] and Action against Hunger (AAH) [31] [Table 1] and identification of the presence of any proxy indicator commonly used in Malawi to indirectly identify individuals infected or affected by HIV/AIDS when blood testing it is not possible or appropriate [32]. Baseline nutritional assessments included weight and height, MUAC, and presence or absence of bilateral, pitting oedema [21,33-35]. Information on the parents' vital status was also obtained.

**Table 1:** Clinical algorithms for diagnosing paediatric HIV

**Algorithm**	**Variables**		**Diagnostic Criteria**
	**History**	**Physical findings**	

**Original IMCI† Algorithm for Paediatric HIV**	• Chest infection requiring hospital admission in the past 3 months	• Weight below 3^rd ^centile	• Classify as suspected symptomatic HIV infection if 3 positive findings
	• > = 2 episodes of diarrhea in the past 3 months	• Poor weight gain (growth monitoring card)	
	• Episode of persistent diarrhea (lasting 14 days) in the past 3 months	• Any enlarged lymph glands in more than one of the following sites: neck, axillary or groin	
	• Fever > = 1 month	• Oral thrush extending to the back of the mouth or throat	
	• Poor appetite		
	• Chronic ear infection (14 days)		
	• History or evidence of past or present herpes zoster		
	• History or evidence of severe seborrheic dermatitis		
	• History of past or present TB		
	• Parent or sibling known to have TB		
	• Parent or sibling known to be HIV-positive		

**Improved IMCI Algorithm (South Africa adaptation)**	• Pneumonia today§	• Weight below 3^rd ^centile	• Classify as suspected symptomatic HIV infection if 3 positive findings
	• History of weight loss¶	• Poor weight gain (history or RTH card)	
	• Persistent diarrhea now or in past 3 months	• Any enlarged lymph glands in more than one of the following; neck, axillae or groin	
	• Ear discharge now or in the past	• Oral thrush	
		• Parotid swelling	

**Action Against Hunger Algorithm**	• Ear discharge now or in the past		• When child presents with > = 3 criteria refer for further HIV support/care and health education
	• Enlarged lymph glands now		
	• Pneumonia today or persistent cough > 1 month		
	• Persistent diarrhea		
	• Low weight gain¶		
	• Oral thrush		
	• Marasmus or Marasmus/Kwashiokor		
	• Fever for more than one month		
	• Child is an orphan (one or both parents)		
	• Child's parents are sick or one of siblings has died		

† IMCI = integrated management of childhood illness § Pneumonia was ascertained by asking if the child had breathing abnormalities (fast breathing, chest in-drawing or nasal flaring) and/or severe cough. ¶ All children were assumed to have a history of weight loss/low weight gain.

### Standardisation and quality control of clinical data

A pilot study to train and standardize the nurses in the collection of clinical data was conducted prior to commencing the RC. The principal researcher (PB), a paediatrician with experience in managing HIV infected children, carried out the training and the standardization process. He also provided daily supervision of the 3 nurses during the first month and twice weekly thereafter. The supervision included examination of 2 randomly selected children per nurse and comparing the paediatrician findings and the nurse findings. Finally, all the data collected by the nurses were checked in the office by the paediatrician for consistency prior to data entry. The 3 trained nurses collected data at enrolment in the RC and admission and follow up data for the PC.

### HIV testing procedures

HIV sero-status in adults and children > 12 months was ascertained via antibody testing from finger-prick blood samples using a serial algorithm with Determine^® ^as first test and Unigold^® ^as a confirmatory test [36,37]. For discordant results, Bioline^® ^rapid test was used as the tie-breaker. For children < 12 months, whole blood HIV DNA polymerase chain reaction (PCR) testing was used (Roche Amplicor Version 1.5). Children between 12 and 18 months were tested using the same algorithm as for older children, but positive antibody results were confirmed by PCR since maternal antibodies may persist in infant blood for up to 18 months [38,39]. Rapid test results were given to caregivers within one hour of the test. PCR test results were provided within 2 weeks. Caregivers' uptake and HIV test results presented in the present papers concern only biological parents of the child.

### Treatment provided

Children were treated using standard CTC protocol. Children with SAM without complications were directly admitted into OTP. Children in both cohorts were provided with Vitamin A, de-worming, anaemia treatment, antibiotics for bacterial infections, and malaria prophylaxis according to standard CTC protocols [33]. For the RC, children found to be anaemic (haemoglobin < 11 g/dl) received the standard Malawian IMCI anaemia treatment and were referred to the nearest health centre for continuation of treatment and follow up. Children with severe anaemia (Haemoglobin < 6 g/dl) were referred to the district hospital for appropriate management. RUTF (200 kcal/kg/day) was provided as weekly take-home rations. During the RC recruitment a protection ration was given to households of admitted children. No protection ration was given during the PC recruitment. All HIV-positive children were referred to the Lighthouse Project in Lilongwe, which provides comprehensive paediatric HIV care following national guidelines. HIV-positive adults were referred to the Dowa District Hospital Antiretroviral Therapy (ART) clinic.

### Statistical analyses

Data analyses were carried out using Epi-Info 6.04 [40] and SPSS for Windows Version 12 [41]. Daily weight gain (g/kg/day) was calculated as the difference between the weight (in grams) at discharge and the weight (in grams) at admission (RC) or lowest weight recorded during participation in CTC (PC), divided by weight at admission (in kilograms) multiplied by number of days in the programme. Changes in MUAC and WFH were determined by calculating the difference between anthropometric measurements at admission and discharge. Means, medians and inter-quartile ranges (IQR) are used to describe and compare continuous parameters. Differences in means were ascertained using Student's t-test and analysis of variance; Kruskall Wallis tests were used to compare medians. Dichotomous variables were compared using χ^2 ^analyses and Fisher's exact test. The sensitivity, specificity, positive predictive value and negative predictive value of different proxy indicators and algorithms for diagnosing paediatric HIV infection were also determined using the PC data. The proxy indicators researched are: presence of a chronically ill adult in the household, recent premature death of an adult on the household, female headed household, widow headed household, elderly headed household, child headed household and presence of a tuberculosis infected child or adult[32]. Stepwise logistic regression with backward elimination was used to identify predictors of HIV infection (α = 0.05).

Written informed consent was obtained from all study caregivers, usually the mother. The study protocol was approved by the College of Medicine Research and Ethics Committee in Malawi.

## Results

**Figure 1:**
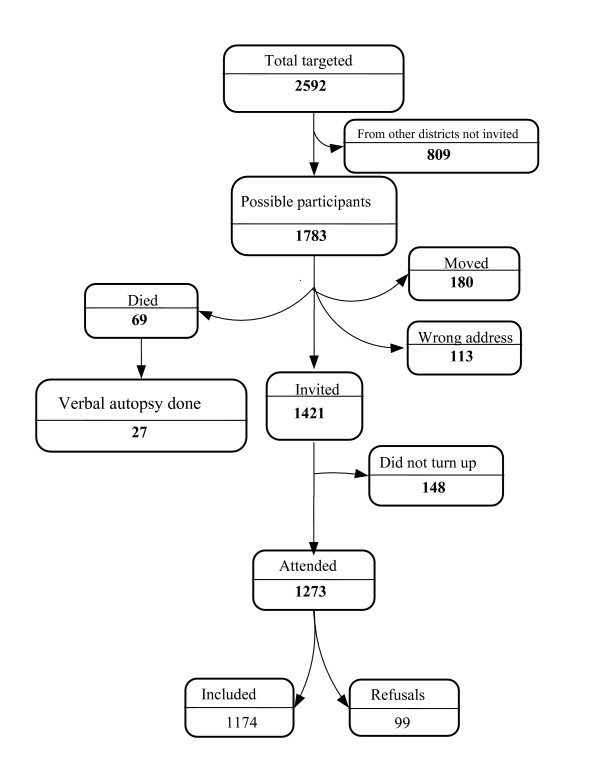
Description of the retrospective cohort.

A total of 2,592 children under 5 years of age had participated in the CTC programme prior to the study. Of these, 809 (31.2%) resided outside the study area and were not eligible for the RC. Of the 1783 possible participants, 180 had moved, 69 had died (according to key informants), 113 could not be located due to an improper address and 1,421 were invited to participate [Figure 1]. The cause of death was ascertained for 27 (39%) of the 69 children that died with the main causes being malaria (n = 8), HIV (n = 7), malnutrition (n = 7), and pneumonia (n = 3).

Of the 1421 children invited to participated, 148 (10.4%) did not turn up. Amongst the returnees, the median time between discharge from the programme and the invitation to participate in the study was 15.6 months (IQR: 10.5–23.3), the average age at CTC admission was 30.0 (17.2) months (median: 26.9; IQR: 17.6 – 37.8) and at study enrolment was 47.2 months (median: 44.3; IQR: 34.4–57. 2). VCT uptake was 92.2% (1174/1273) for children and 58.4% (743/1273) for caregivers. The reasons for refusing the HIV test were: need to consult with husband in 15.2% (15/99) of cases, not psychologically ready in 13.1% (13/99) of cases, fear of the results in 8.1% (8/99), no authority for allowing testing in 7.1% (7/99), not needed because the child is healthy in 7.1%(7/99), already tested in 5.1% (5/99) and others (believes already infected or fear of aggravating child anaemia or not willing to cause pain) in 2.0% of cases (3/99). Thirty-eight caregivers (38.4% of refusals) did not disclose the reasons for refusal.

735 children were eligible for the PC. The average age at admission was 26.5 (13.7) months (median: 23.0; IQR: 16.9–34.1). VCT uptake was 97% (714/735) for children and 64.1% (471/735) for parents in this cohort. The reasons for refusal were not collected by study design.

There was no difference in socio-demographic and clinical characteristics at admission between tested and not tested children in either the RC or the PC (data not shown).

Other baseline characteristics of children in the RC and PC groups are shown in Table 2. Family history of tuberculosis (TB) was more common RC (p < 0.01). Only 22 PC children (3.1%) and 29 RC children (2.5%) were HIV-infected (p = 0.45). HIV prevalence was similar amongst parents in both cohorts. Amongst caregivers tested, 5.4% (58/1081) of mothers and 2.9% (3/133) of fathers were HIV-infected giving an overall prevalence amongst caregivers of 5.0% (61/1214).

**Table 2:** Baseline characteristics of participants in the retrospective and prospective cohorts

	**Prospective**			**Retrospective**			**Total**	
	**n**	**%**	**mean (SD)**	**n**	**%**	**mean(SD)**	**N**	**%**

**VCT uptake**								
Accepted VCT	714	97.1		1174	92.2		1888	94.0
Refused VCT	21	2.9		99	7.8		120	6.0
**Total**	735	100.0		1273	100.0		2008	100.0
**Parents testing uptake**								
Parent and child tested	471	66.0		743	63.3		1214	64.3
Only child tested	243	34.0		431	36.7		674	35.7
**Total**	**714**	**100.0**		**1174**	**100.0**		**1888**	**100.0**
**Parents' vital status**								
Orphan (1 or both parents dead)	35	5.0		100	8.6		135	7.3
Both parents alive	661	95.0		1065	91.4		1726	92.7
**Total**	696	100.0		1165	100.0		1861	100.0
**Family history of TB**								
Yes	65	9.1		127	19.1		192	13.9
No	648	90.9		539	80.9		1187	86.1
**Total**	713	100.0		666	100.0		1379	100.0
**Sex**								
Female	353	49.4		595	52.4		948	51.3
Male	361	50.6		540	47.6		901	48.7
**Total**	714	100.0		1135	100.0		1849	100.0
**Age (months)**								
< 12	62	10.5		157	16.8		219	14.4
12-<24	272	46.1		354	37.9		626	41.1
24-<36	153	25.9		275	29.5		428	28.1
> = 36	103	17.5		147	15.8		250	16.4
**Total**	590	100.0		933	100.0		1523	100.0
**Admission category**								
Oedema	597	83.7		659	67.4		1256	74.3
Maramus	51	7.2		160	16.4		211	12.5
MUAC < 110 mm	46	6.5		75	7.7		121	7.2
Other criteria	19	2.7		84	8.6		103	6.1
**Total**	713	100.0		978	100.0		1691	100.0
**Presence of oedema**								
Yes	597	83.7		659	67.4		1256	74.3
No	116	16.3		319	32.6		435	25.7
**Total**	713	100.0		978	100.0		1691	100.0
**WHM % (SD)**			83.3(13.0)			84.2(14.4)		
> = 80	397	58.7		552	59.9		949	59.4
70 to < 80%	187	27.6		186	20.2		373	23.3
< 70%	93	13.7		183	19.9		276	17.3
**Total**	677	100.0		921	100.0		1598	100.0
**MUAC (SD)**			118.0(16.9)			119.2(18.9)		
> = 125 mm	219	34.9		326	42.0		545	38.8
110 to < 125 mm	223	35.5		208	26.8		431	30.7
< 110 mm	186	29.6		242	31.2		428	30.5
**Total**	628	100.0		776	100.0		1404	100.0

In both RC and PC cohorts, there was no significant difference in the sex ratio, age distribution and in frequency of family history of tuberculosis among HIV-infected and HIV-negative children (Table 3). HIV-infected children were more likely to be orphaned and to come from a household with at least one HIV proxy indicator (Table 3). Nutritional status at admission differed between HIV-infected and HIV-uninfected children (Table 4). HIV-infected children were more likely to be admitted with MUAC < 110 mm and less likely to have oedema than uninfected children in both cohorts. Oedematous malnutrition, however, was the most frequent admission characteristic in both infected and uninfected children. An equal proportion of HIV-positive (8/22 = 36.4%) and HIV-negative children (249/692 = 36.0%) required inpatient stabilisation prior to referral to OTP (p = 0.971). About one-third of all HIV-positive children were orphaned (at least one parent dead) compared with < 10% of HIV-negative children (p < 0.001 in both cohorts).

**Table 3:** Demographic characteristics, tuberculosis history and nutrition admission criteria according to the HIV status of children from retrospective and prospective cohorts.

	**Prospective**				**p-value**	**Retrospective**				**p-value**
				
	**HIV+ve**		**HIV-ve**			**HIV+ve**		**HIV-ve**		
**Variable**	**n**	**%**	**n**	**%**		**n**	**%**	**n**	**%**	
**Sex**										
Female	12	54.5	341	49.3		15	53.6	580	52.4	
Male	10	45.5	351	50.7	0.626	13	46.4	527	47.6	0.902
**Total**	22	100.0	692	100.0		28	100.0	1107	100.0	
**Age**										
< 12	5	25.0	57	10.0		4	16.0	153	16.9	
12-<24	6	30.0	266	46.7		9	36.0	345	38.0	
24-<36	4	20.0	149	26.1	0.099	8	32.0	267	29.4	0.993
> = 36	5	25.0	98	17.2		4	16.0	143	15.7	
**Total**	20	100.0	570	100.0		25	100.0	908	100.0	
**Parents status**										
Orphan (1 or both parents)	7	31.8	28	4.2		10	34.5	90	7.9	
Parents alive	15	68.2	646	95.8	< 0.001	19	65.5	1046	92.1	< 0.001
**Total**	22	100.0	674	100.0		29	100.0	1136	100.0	
**Presence of proxy indicator**										
> = 1 proxy present	14	66.7	138	21.5		15	53.6	261	23.9	
None	7	33.3	505	78.5	< 0.001	13	46.4	832	76.1	< 0.001
**Total**	21	100.0	643	100.0		28	100.0	1093	100.0	
**Family history of tuberculosis**										
Yes	4	19.0	54	8.0		7	29.2	120	18.7	
No	17	81.0	621	92.0	0.089	17	70.8	522	81.3	0.193
**Total**	21		675	100.0		24	100.0	642	100.0	
**Admission category**										
oedema	14	63.6	583	84.4		16	55.2	643	67.8	
No oedema	8	36.4	108	15.6	0.017	13	44.8	306	32.2	0.017
**Total**	22	100.0	691	100.0		29	100.0	949	100.0	

**Table 4:** Nutritional status at enrolment and the impact of CTC in HIV-positive and HIV-negative children

	**HIV positive**			**HIV negative**			**P-value**
			
	**n**	**%**		**n**	**%**		
**Prospective cohort**							
**Admission category**							
Oedema	14	63.6		583	84.4		0.017†
Maramus	1	4.5		50	7.2		
MUAC < 110 mm	5	22.7		41	5.9		
Others criteria	2	9.2		17	2.5		
**Total**	22	100.0		691	100.0		

**WHM% mean(SD)**			80.5(8.9)			83.3(12.6)	0.3
> = 70%	20	90.9		564	86.1		0.755
< 70%	2	9.1		91	13.9		
**Total**	22	100.0		655	100.0		

**MUAC mean (SD)**			109.2(16.4)			118.3(16.9)	0.025
> = 110 mm	7	38.9		435	71.3		0.003
< 110 mm	11	61.1		175	28.7		
**Total**	18	100.0		610	100.0		

**CTC Outcomes**							
Recovered	13	59.1		523	83.4		0.002
Defaulted	5	22.7		89	14.2		< 0.001
Died	4	18.2		11	1.8		< 0.001
Transfer or still in programme				4	0.7		-
Median weight gain (IQR) *g/kg/day*	20		2.8 (1.3–3.9)	614		4.7(2.9–6.7)	0.007
Median MUAC change (IQR) *mm/day*	9		0.11(-0.03–0.31)	361		0.21(0.05–0.39)	0.223
Median LoS (IQR) *days*	20		56(36–68)	622		42(28–63)	0.25

**Retrospective cohort**							
**Admission category**							
Oedema	16	55.2		643	67.8		0.154†
Marasmus	6	20.7		154	16.2		
MUAC < 110 mm	5	17.2		70	7.4		
Others criteria‡	2	6.9		82	8.6		
**Total**	29	100.0		949	100.0		

**WHM% (SD)**			81.0(15.7)			84.3(14.4)	0.26
> = 70	17	68.0		721	80.5		0.130
< 70%	8	32.0		175	19.5		
**Total**	25	100.0		896	100.0		

**CTC Outcomes**							
Median Weight gain (IQR) *g/kg/day*	24		2.2 (1.6–4.0)	880		3.1(1.1–5.9)	0.309
Median MUAC change (IQR)*mm/day*	11		0.22(0.01–0.45)	476		0.25(0.03–0.48)	0.891
Median LOS (IQR) *days*	25		63(42–128)	912		42(28–67)	0.002

† Comparison % oedematous malnutrition; ‡ others criteria = age above 6 months and weight less than 4 kg, child with visible wasting but not meeting marasmus and MUAC criteria and less than 6 months children.

Nutritional recovery also varied by HIV status (Table 4). In the PC, 13 HIV-infected children (59.1%) and 523 HIV-uninfected (83.4%) achieved discharge WFH (p = 0.003). Average rate of weight gain was 4.7 g/kg/day in uninfected children and 2.8 g/kg/d in HIV-positive PC children. Median recovery time to discharge was 42 days for uninfected children and 56 days for HIV-positive children. Five HIV-positive children (22.7%) and 89 HIV-negative children (14.2%) defaulted from the programme (p < 0.001). Four HIV-positive children died during the CTC treatment (18.2%); mortality amongst uninfected children was 1.8% (p < 0.001). Estimated daily weight gain was lower in the RC children, in part due to the high prevalence of oedema at admission and use of admission weight to estimate this parameter.

Nutritional status at follow-up varied according to HIV status. Approximately 15 months after discharge, 24 out of 28 (85.7%) HIV-infected RC children were not malnourished (WFH > 80% reference median and no bilateral pitting oedema). However, six of these children had a MUAC below 125 mm, including one with a MUAC < 110 mm, giving an overall malnutrition rate of 35.7% (10 out of 28) in HIV-infected children compared with a malnutrition rate of 2.0% (22/1094) in HIV-negative children (p = 0.001).

The predictive characteristics for variables included in clinical algorithms, for individual proxy indicators, and for the 3 algorithms presently used to diagnose paediatric HIV are shown in Table 5. All three of the clinical algorithms had positive likelihood ratio lower than 10 and negative likelihood ratio higher than 0.1 suggesting limited utility for ruling-out and ruling in paediatric HIV. The presence of one or more HIV proxy indicators in combination with MUAC < 110 mm was also a poor predictor of the presence of an HIV infection. In contrast, however, severely malnourished children with a MUAC > 110 mm and no proxy indicators were 10-fold less likely to be HIV-infected, suggesting that this combination may be useful for ruling out HIV where confirmatory paediatric testing is not widely available. The other criteria with high positive likelihood for predicting HIV (i.e., PLR > 10) are having a deceased father or living in a widow-headed household.

**Table 5:** The prevalence and diagnostic characteristics of proxy indicators and clinical algorithms for identifying HIV infection in severely malnourished children

	**Prevalence**	**OR**	**Sensitivity **	**Specificity **	**PPV†**	**NPV‡**	**PLR$**	**NLR&**
	**(%)**	**(95%CI)**	**(%)**	**(%)**				
**Individual proxy indicators, symptoms and signs**								
								
From widow headed household	1.4	30.0(7.4–121.6)	19	99.2	44.4	97.4	23.8	0.8
Looked after by grand-mother	4.1	4.1(1.1–14.6)	13.6	96.3	10.7	97.1	3.7	0.9
Orphan (one or both parents dead)	5.0	10.8(4.1–28.5)	31.8	95.8	20	97.7	7.6	0.7
From female headed household	7.1	5.8(2.1–17.2)	28.6	93.6	12.8	97.6	4.5	0.8
**Symptoms and signs used in algorithms**								
Child with tuberculosis	1.4	9.2(1.8–47.7)	10	98.8	20	97.4	8.3	0.9
Minor muco-cutaneous manifestations	7.2	4.6(1.6–13.2)	25	93.3	10	97.6	3.7	0.8
**Variables associated with HIV in the present study**								
Death of the father	3.1	15.6(4.7–49.9)	27.3	97.6	27.3	97.6	11.4	0.7
Age < 12 months or age > 59 months	13.6	3.7(1.4–9.5)	35	85.2	8.8	97.5	2.4	0.8
MUAC < 110 mm	29.6	3.5(1.9–10.2)	61.1	71.3	5.9	98.4	2.1	0.5
Absence of oedema	17.1	2.9(1.1–7.7)	36.4	83.7	8.3	93	2.2	0.8
Axillary nodes enlargement	4.8	6.7(1.7–26.4)	23.1	95.7	13.6	97.7	5.4	0.8

**Algorithm and combinations**								
South-African IMCI modified algorithm for paediatric HIV diagnosis			20.0	94.5	8.7	97.8	3.6	0.8
Original IMCI algorithm			9.1	96.7	8.0	97.1	2.8	0.9
Action Against Hunger algorithm			60.0	62.1	3.5	98.3	1.6	0.6
Presence one or more proxy indicators and MUAC < 110 mm			95.5	54.5	7.1	99.7	2.1	0.1

† PPV = positive predictive value; ‡ NPV = Negative predictive value; § PLR = likelihood ratio for a positive test; & NLR = likelihood ratio for a negative test

## Discussion

The study aims were to assess whether a CTC programme can be used as an entry point for HIV services, including HIV testing and treatment of malnutrition in HIV-positive children, and to compare outcomes of HIV-positive and HIV-negative children within the programme. More than half of the HIV-infected children in the PC (59.1%) recovered to a satisfactory nutritional status using CTC protocols, suggesting that SAM can be managed in the community for many HIV-infected children. Furthermore, about two-thirds of the infected children identified after discharge were still adequately nourished. This nutritional recovery occurred without use of antiretroviral therapy (ART), suggesting that severe malnutrition was primarily the result of semi-starvation among recovering study children. These findings are different from those normally observed in developed and middle income countries where severe malnutrition in HIV-positive children is typically caused by HIV-related metabolic disturbances, which do not improve without ART [42]. Indeed, the poor response to nutritional intervention of cachexia, a form of malnutrition that is primarily due to chronic systemic inflammation is a well-known phenomenon [43,44].

The 59.1% recovery rate for HIV-infected children observed in the PC arm of our study includes deaths in both the inpatient-based stabilisation phase and the outpatient-based recovery phase of care. This figure is similar to the 56% recovery rate reported in a study in southern Malawi where RUTF was used for Home Therapy (HT) in the recovery phase of care, after patients had been discharged from a hospital Nutrition Rehabilitation Unit (NRU) [18]. As mortality amongst severely malnourished HIV-positive children is usually highest during the initial phase of treatment and may exceed 30% [45], the results from our study are encouraging.

It is possible that our improved recovery rates arise from the decentralised nature of the CTC model of care that is designed to remove barriers to access and promote early presentation before serious complications develop. However, the numbers of HIV subjects in our PC are small and these findings need to be confirmed by larger studies. The results of the present study are also encouraging when compared with the 3-month mortality rate of 42.9% among severely wasted (WFH < 70%) children started on ART, recently reported in Malawi [46]. Mortality in this group was > 10-fold higher than among children starting on ART who were not acutely malnourished (WFH > 80%) [46].

As observed in other nutritional studies carried out in Malawi, the HIV-positive children in our cohorts recovered more slowly than the HIV-negative children [18,45]. The possible reasons for slower weight gain include reduced intake due to poor appetite, nutrient malabsorption, increased incidence of infections that were unresponsive to the broad-spectrum antibiotics used, and increased nutrient requirements due to HIV [47]. Despite the possibility of reduced appetite especially at the beginning of treatment, we believe that HIV-positive children may need more RUTF than HIV-negative children to achieve similar growth rates and improvements in other nutritional indices. Increasing the amount of daily energy offered to HIV infected children may improve their weight gain and reduce the length of stay in the program, and further testing of this hypothesis is needed. Continued nutritional surveillance and supplementation after discharge may also help HIV-infected children to remain well-nourished. Reducing recovery time and subsequent length and cost of program participation will reduce default rates, which occurred, on average, after 56 days by families with all HIV-positive children but at 70 days for those who finally defaulted. Similarly, adapting CTC routine antibiotic treatment to the epidemiology of HIV-associated infections and inclusion of routine prophylactic cotrimoxazole for HIV-positive children, as currently recommended by WHO, may improve recovery in this group [1,48].

The low relapse rates following CTC is in contrast to NRU and other outpatient SAM treatment programmes where morbidity and mortality after discharge are high [49-51]. Although survival bias cannot be ruled out as an explanation for this finding, it is possible that the CTC design, which uses community mobilization and referral for early identification and treatment of SAM also improves long-term recovery compared to hospital-based treatment programmes. SAM is a progressive condition and prognosis is directly associated with the lead time to presentation and treatment. Initiating nutritional intervention as soon as SAM presents may be especially important for HIV-infected and exposed children.

The high VCT uptake for adults and children, the low HIV-prevalence amongst SAM children, and the low nutritional relapse rate amongst surviving HIV-positive and uninfected children over a year after discharge are all noteworthy in our study. The high VCT uptake is comparable to that observed recently in some NRUs in Malawi [16] but contrasts with anecdotal reports of reluctance to come forward for HIV testing offered by clinics and therapeutic feeding programmes. We believe that the "opt-out" approach used in this study, with HIV testing offered to everyone with the right to refuse rather than the standard "opt-in" approach where people have to specifically request HIV testing, contributed to the high uptake seen here [11,52,53]. We also believe that offering testing through a programme such as CTC that is well established in the community improves trust and reduces the fear of stigmatization. Caregivers were informed about the opportunity for HIV testing one week prior to travelling to the health centre and no substantial compensation was offered, ruling out the likelihood of coercion in the RC. The high uptake observed here suggests that CTC is a potentially innovative way to increase access to and coverage of HIV testing, particularly in rural areas [54]. It is important to note however that CTC would have to be combined with other community based HIV testing and counselling approaches like the home based and mobile VCT in order to obtain good coverage; in prior studies we observed that only 16% of HIV-affected households has a malnourished child treated in the CTC programme in the past 18 months [9,55,56].

There are a number of factors that are likely to have contributed to the low prevalence of HIV amongst severely malnourished children in this study. Chronic food insecurity, frequent common childhood illnesses, poor access to modern health care and suboptimal complementary feeding practices all cause SAM in the absence of HIV in Malawi [57-59]. The decentralised nature and the high coverage rates obtained by CTC programmes means that a higher proportion of admissions live in remote rural areas far from towns and main roads [60] in contrast to admissions in more centrally located urban hospital units. People from rural areas are all subsistence farmers, have poor income and low educational level and have no possibility for travelling within or outside the country. These factors are known to increase the prevalence of malnutrition and lower that of HIV [57,61-64]. Lastly, the low HIV prevalence might also be explained by the high mortality of HIV infected infants biasing our data to include those children who have survive infancy. Without effective treatment, it is estimated that over 50% of infants who acquired HIV infection through mother-to-child transmission will die by two years of age (compared to 8% of uninfected children) while in Malawi kwashiorkor, the most common form of SAM in children, peaks between 18 to 23 months of age [65-68]. The relatively older average ages of children in both the PC (26.4 months) and RC cohorts (47.2 months), suggests the possibility of survival bias.

Our estimated adult HIV prevalence of 5.0% is predictably lower than the antenatal HIV prevalence rate in the central region (9.8%) but similar to comparable adult prevalence rates of 6.4% and 4.1% for the region and for an adjacent district, respectively, as reported in the 2004 Malawi Demographic and Health Survey [69,70]. Our study suggests that targeting adult caretakers of malnourished children for HIV testing is feasible but additional outreach and counseling efforts may be needed to increase uptake.

Our analysis confirms that clinical algorithms designed to diagnose paediatric HIV are neither sufficiently sensitive nor specific in severely malnourished children and that blood tests are therefore required to confirm the diagnosis [71]. In context where blood tests are not available, the combination of MUAC > 110 mm and absence of proxy indicators can be used to rule out the presence of HIV. These family history variables could be incorporated into future CTC protocols for confirmed or suspected paediatric HIV in setting without possibility of blood tests [72].

SAM is one of several criteria for initiating ART in HIV-positive children [73]. The fact that some previously undiagnosed HIV-positive children recovered from malnutrition, and were still healthy and asymptomatic an average of 15 months after discharge from CTC without ART, suggests that the presence of malnutrition should not be the sole criteria for initiating ART in food insecure settings. One possibility is that initiation of ART could be reserved for children who do not respond to CTC or at least could be delayed until nutrition improvement to minimize antiretroviral side effects. Several studies have reported that HIV-infected children tend to develop marasmus rather than kwashiorkor and that CD4 count remains higher in HIV-infected children with kwashiorkor compared to those with marasmus [17,74,75]. Therefore, delayed initiation of ART could be considered for children with kwashiorkor. This approach could help to prevent unnecessary exposure to ARV drugs that have side effects and toxicity and to reduce the risk of developing resistance [76]. Further research, probably in the form of randomized trials, is urgently needed to strengthen this evidence base before any change of practice is recommended.

Several limitations of our study deserve mention. This study was carried out in conjunction with an ongoing CTC programme and clinical records were reviewed in order to obtain data on nutritional recovery. Although programme procedures were standardized, we were unable to verify nutritional measurements for accuracy. As noted previously, the RC may be subject to survival bias, and therefore we have limited our use and interpretation of the RC data. The statistical power of these analyses is also limited by the small number of HIV-positive children in the study and by further reduction of the sample size due to missing data for age and nutritional status. Nevertheless, the data from both cohorts paint a consistent picture with regard to the potential positive impact of Community-based Therapeutic Care for managing SAM in HIV-positive and uninfected children in rural Africa.

## Conclusion

The results of the present study demonstrate that CTC is a valuable entry point for HIV testing for severely malnourished children and that good recovery rates can be achieved in HIV-infected severely malnourished children admitted to the program. These results indicate that CTC can be used to improve the coverage of HIV services, especially in rural areas. The approach has several important advantages over traditional inpatient therapeutic care, including earlier intervention, greater coverage, and increased accessibility. All of these characteristics are particularly important for providing timely care to HIV-exposed and vulnerable children. Additional research on feeding protocols for HIV-infected children and on timing of ART initiation are needed to refine CTC protocols in settings where HIV is common.

## Competing interests

The authors Paluku Bahwere, Kate Sadler, Saul Guerrero and Steve Collins work for Valid International, an organisation that has been engaged in the research and development of Community-based Therapeutic Care. Dr Steve Collins is also unpaid director of Valid Nutrition, a not-for-profit company established to research and manufacture ready-to-use therapeutic food in developing countries. There is no conflicting interest for all the other authors.

## Authors' contributions

PB: concept and design of the research, data collection, data analysis and interpretation, drafting of the paper. EP: concept and design of the research, critical revision of paper content. MCJ: data collection, analysis of data and revision of the manusdcript. KS: concept and design of the research, data analysis and interpretation, revision of the draft of the paper. CHGT: concept and design of the research, critical revision of paper content. SG: design and concept of the research, data collection and revision of the manuscript. SC: concept and design of the research, data analysis and interpretation, revision of the draft of the paper. All authors read and approved the final manuscript.

## Pre-publication history

The pre-publication history for this paper can be accessed here:


